# Genetic diversity and natural selection of *Plasmodium vivax* reticulocyte invasion genes in Ecuador

**DOI:** 10.1186/s12936-023-04640-0

**Published:** 2023-08-03

**Authors:** Andrés Núñez, Francis B. Ntumngia, Yasel Guerra, John H. Adams, Fabián E. Sáenz

**Affiliations:** 1 Centro de Investigación para la Salud en América Latina, Facultad de Ciencias Exactas y Naturales, Pontificia Universidad Católica del Ecuador, Quito, Ecuador; 2 Center for Global Health and Interdisciplinary Research, College of Public Health, University of South Florida, FL Tampa, USA; 3 Grupo de Bio-Quimioinformática, Universidad de Las Américas, Quito, Ecuador

**Keywords:** *Plasmodium vivax*, Ecuador, Genetic diversity, Merozoite invasion, Reticulocytes, Natural selection

## Abstract

**Background:**

Knowledge of the diversity of invasion ligands in malaria parasites in endemic regions is essential to understand how natural selection influences genetic diversity of these ligands and their feasibility as possible targets for future vaccine development. In this study the diversity of four genes for merozoite invasion ligands was studied in Ecuadorian isolates of *Plasmodium vivax*.

**Methods:**

Eighty-eight samples from *P. vivax* infected individuals from the Coast and Amazon region of Ecuador were obtained between 2012 and 2015. The merozoite invasion genes *pvmsp-1-19*, *pvdbpII*, *pvrbp1a-2* and *pvama1* were amplified, sequenced, and compared to the Sal-1 strain. Polymorphisms were mapped and genetic relationships between haplotypes were determined.

**Results:**

Only one nonsynonymous polymorphism was detected in *pvmsp-1-19*, while 44 nonsynonymous polymorphisms were detected in *pvdbpII*, 56 in *pvrbp1a-2* and 33 in *pvama1.* While haplotypes appeared to be more related within each area of study and there was less relationship between parasites of the coastal and Amazon regions of the country, diversification processes were observed in the two Amazon regions. The highest haplotypic diversity for most genes occurred in the East Amazon of the country. The high diversity observed in Ecuadorian samples is closer to Brazilian and Venezuelan isolates, but lower than reported in other endemic regions. In addition, departure from neutrality was observed in Ecuadorian *pvama1*. Polymorphisms for *pvdbpII* and *pvama1* were associated to B-cell epitopes.

**Conclusions:**

*pvdbpII* and *pvama1* genetic diversity found in Ecuadorian *P. vivax* was very similar to that encountered in other malaria endemic countries with varying transmission levels and segregated by geographic region. The highest diversity of *P. vivax* invasion genes in Ecuador was found in the Amazonian region. Although selection appeared to have small effect on *pvdbpII* and *pvrbp1a-2, pvama1* was influenced by significant balancing selection.

**Supplementary Information:**

The online version contains supplementary material available at 10.1186/s12936-023-04640-0.

## Background

Malaria has been a major public health problem worldwide. In 2021, an estimated 247 million cases of malaria occurred, and 619,000 deaths were reported globally. In the Americas, malaria is primarily distributed in the Southern hemisphere and *Plasmodium vivax* is the predominant parasite, representing 64% of all malaria cases [1]. Ecuador has effectively managed the spread of malaria in recent years. The diagnosed prevalence of the disease has decreased by approximately 99% in the last 15 years. Transmission of malaria is mostly periodic in the coastal region and perennial in the Amazon, mainly rural and affects mostly men in working age [2]. Periodic outbreaks occur in endemic regions and since 2015 the number of reported malaria cases increased each year, reaching 2,190 in 2021, out of which 88% corresponded to *P. vivax* [2]. However, it is likely that the real number of infected individuals is underestimated since there are continuous undocumented reports of vivax malaria, and several studies have shown that asymptomatic and submicroscopic infections are common in Ecuadorian endemic communities [3, 4].

*Plasmodium vivax* merozoites preferentially infect human reticulocytes in a dynamic multiple-step process progressing rapidly from initial attachment to apical orientation and junction formation, then invasion and membrane sealing [5]. Merozoite surface protein 1 (MSP1) is the most abundant protein on the parasite surface and is implicated in the invasive merozoite’s initial attachment [6]. The mature form of MSP1 has undergone proteolytic processing and exists on the merozoite surface as a complex of four peptide fragments of 83, 30, 38 and 42 kDa [7–9]. At the time of invasion, the 42 kDa fragment is further processed in two more fragments of 33 and 19 kDa. The latter, MSP1_19_ (coded by *pvmsp-1-19*) remains on the merozoite surface and is carried into the invaded reticulocyte [10, 11]. The EGF-like domains and disulfide binding pattern of MSP1_19_ are highly conserved and several studies demonstrated that it is highly immunogenic [12, 13]. Additionally, it has been shown that antibodies against MSP1_19_ can significantly inhibit parasite growth in vitro and consequently anti-MSP1 immunity is associated with immune protection against malaria [14–16].

In the next steps of invasion, merozoites orient their apical end towards the reticulocyte surface membrane that leads to junction formation. This process involves proteins released from the organelles of the apical complex, including Duffy binding protein (DBP) and Reticulocyte binding protein 1a (RBP1a). These proteins are synthesized during the final stages of asexual development and then sequestered in the micronemes until transported to the surface of the parasite in the moments of invasion [17, 18]. Region II (DBPII) contains the receptor-binding motifs necessary for adherence to its cognate receptor, the Duffy antigen receptor for chemokines (DARC) [19–21]. Although less immunogenic than MSP1, antibodies directed against PvDBPII reduce binding efficiency to DARC and inhibit invasion of *P. vivax* merozoites into reticulocytes [22]. RBP1a has been suggested to play an important role in binding and re-orientation of merozoites when invading reticulocytes [23]. Although PvRBP1a has been well characterized in the last few years, the specific binding site is still under discussion and the binding motif appears to be between residues 361 and 599, corresponding to *pvrbp1a-2* gene fragment [23, 26].

In junction formation, merozoites form an irreversible attachment as a tight junction with the reticulocyte, which is mediated by another microneme protein, Apical Membrane Antigen 1 (AMA1). Like DBP, AMA1 is transported to the merozoite surface after trafficking through the micronemes where it is processed prior to being transported to the parasite surface during invasion [27]. Once this antigen is on the surface, it can bind to RON2 (Rhoptry Neck Protein 2), which is previously synthesized in the rhoptries of the same merozoite, and then inserted into the surface of reticulocytes to fulfill the self-anchoring function [28, 29]. The extracellular region of AMA1 consists of three structural domains defined by a highly conserved pattern of disulfide bounds. Analysis of the three-dimensional structure of this ligand, revealed that domains I and II belong to the PAN module superfamily, which is involved in binding to receptors in reticulocytes [30]. In addition, several studies suggest that the immunogenic N-terminal extracellular domains I and II that bind RON2 are the targets of inhibitory antibodies, making it an important vaccine candidate against *P. vivax* [31].

In the final phase of invasion, several factors stimulate the actin-myosin motor to power the junction to move from the anterior to the posterior end of the merozoite, bringing the parasite into the reticulocyte while a parasitophorous vacuole is formed around it. Finally, the membrane seals to enclose the parasite inside a vacuole in the reticulocyte [32].

The knowledge about strain variation of these critical merozoite invasion ligands is important since these are targets of protective immunity and potential vaccine candidates. There are some insights about the genetic diversity of *dbpII* in *P. vivax* populations from Colombia [33] and Brazil [34], but the data about allelic variation of these ligands is generally limited in the Americas and the general levels of geographic diversity remain poorly understood. In Venezuela a comparative study of *ama1* diversity among *Plasmodium falciparum* and *P. vivax* was carried out with Amazonian isolates [35]. Also, a recent analysis of *ama1* sequences in Brazilian *P. vivax* isolates determined the implications of haplotype diversity in the immune response [36]. However, only one study, carried out in parasites from southern Mexico, analysed the main genes involved in the invasive process together (*pvmsp1-42*, *pvdbpII* and *pvama1*). The findings revealed some differences in the genetic diversity may occur by region and possible evolutionary forces may influence each gene, depending on its role in reticulocyte invasion [37].

To address this important knowledge gap of these vaccine candidates, studies are needed to provide essential information about the diversity of these genes, their distribution in endemic countries, as well as the influence of evolutionary forces in the generation and maintenance of the genetic variability. Though a relatively small country, the geography of Ecuador is ideally suited to analyse parasite allelic diversity of geographically distinct regions, since the malaria endemic areas are separated by the Andes Mountains, where there are no mosquito vectors for transmission, into a western Pacific region and an Amazonian region.

The aim of this study was to determine the allelic diversity of four *P. vivax* merozoite invasion proteins considered potential candidate vaccines (*pvmsp1-19*, *pvdbpII, pvrbp1a-2* and *pvama1)*, and how natural selection influences this diversity in Ecuador. These findings will help contribute to the feasibility of use of these genes as potential targets in future vaccine development for the region and the country.

## Methods

The study protocol was approved by the Ethical Review Committee of Pontificia Universidad Católica del Ecuador Approval number (#CBE-016-2013). Written informed consent (IC) was provided by study participants and/or their legal guardians for sample collection.

### *Plasmodium vivax* blood samples

**Fig. 1:**
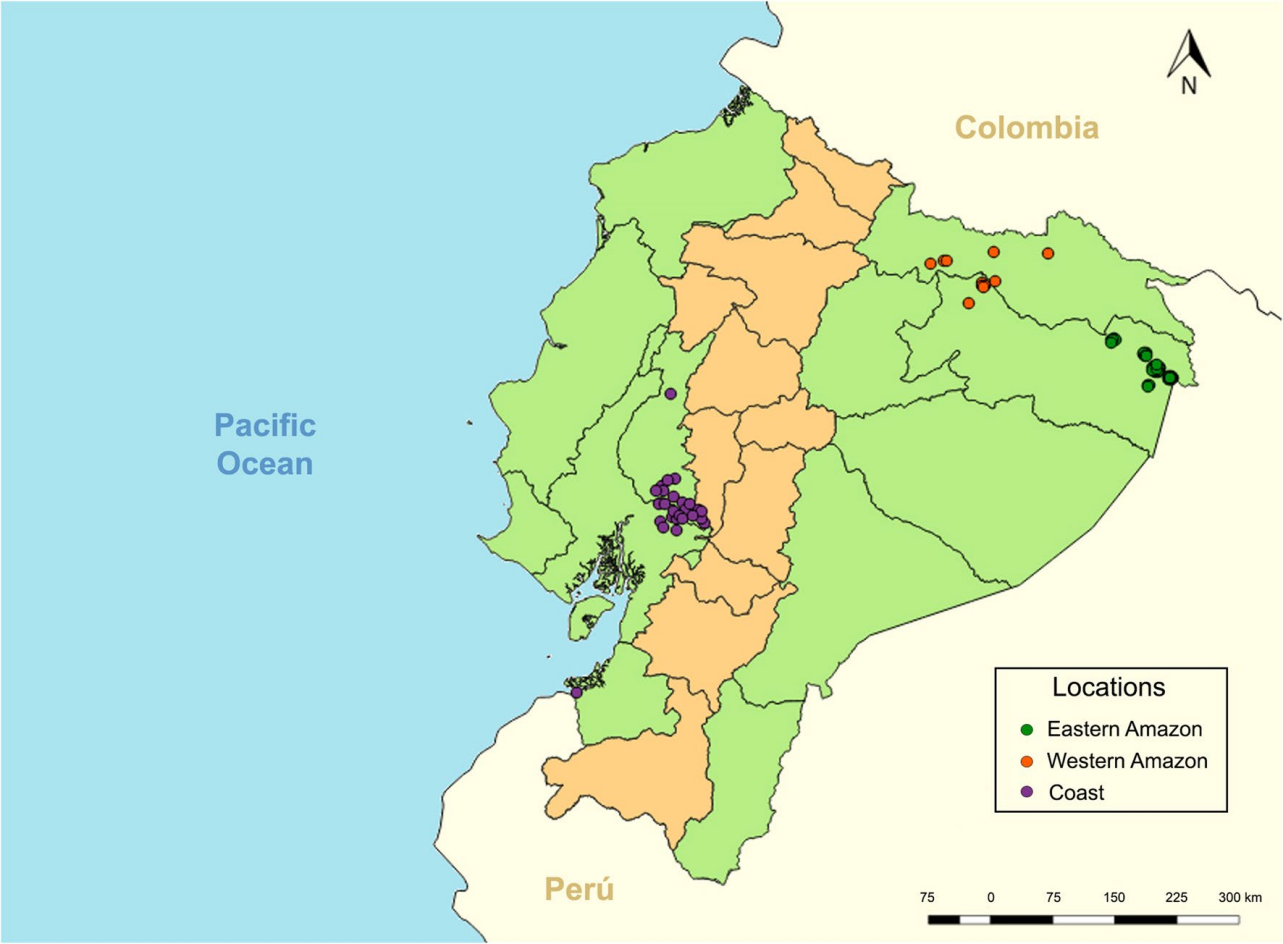
Geographical distribution of the localities assigned for the study. A total of 87 *P. vivax* samples collected between 2012–2015 were used. Localities were assigned depending on the geographical position. Eastern Amazon (green), western Amazon (orange) and coast (purple)

A total of 87 samples from 2012 to 2015 were analysed from individuals diagnosed with *P. vivax* infection by microscopy and PCR [38]. Samples were grouped by their geographic origin: Coast (n = 27); Western Amazon (n = 11); and Eastern Amazon (n = 49) (Fig. 1). Total genomic DNA (gDNA) was extracted from 200 ul of infected blood samples using QIAamp DNA minikit (Qiagen, USA) and resuspended in 60 ul, following the manufacturer’s instructions.

### PCR amplification and DNA sequencing

A *pvmsp1-19* gene fragment of approximately 326 bp was amplified by PCR using primers: *pvmsp1-19F* 5’-ATGAGCTCCGAGCACACATGTATAG-3’ and *pvmsp1-19R* 5’-ATGCACAGGAGGAAAAGCAACATG-3’. The final volume of 37.5 µL contained: 1X GoTaq Colorless Mastermix (Promega, Madison, USA), 25 mM of MgCl_2_, 10 µM of each primer and 3 µL DNA. The PCR cycle was 94 ºC for 2 min, followed by 35 cycles of 94 ºC for 30 s, 62 ºC for 30 s and 72 ºC for 90 s, and a final extension at 72 ºC for 5 min.

A *pvdbpII* gene fragment of approximately 1200 bp was amplified using the primers: *pvdbpIIF* 5’-GATAAAACTGGGGAGGAAAAAGAT-3’ and *pvdbpIIR* 5’-CTTATCGGATTTGAATTGGTGGC-3’ [37]. The PCR was prepared as above, and the PCR cycle was at 94 ºC for 2 min, followed by 35 cycles of 94 ºC for 30 s, 62 ºC for 30 s and 72 ºC for 90 s, and a final extension at 72 ºC for 5 min.

A *pvrbp1a-2* gene fragment of approximately 1064 bp was amplified using the primers: *pvrbp1a-2F* 5’-AATACAATGCGCAAGAATTTTATATC-3’ and *pvrbp1a-2R* 5’-GTGAAGTACACACTTGATTTCCC-3’. The PCR was prepared as above, and the PCR cycle was at 94 ºC for 2 min, followed by 35 cycles of 94 ºC for 30 s, 54.7 ºC for 30 s and 72 ºC for 90 s, and a final extension at 72 ºC for 5 min.

The *pvama1* CDS of approximately 1600 bp was amplified by a nested PCR using the primers: nest 1 *pvama1_F16* 5’-GCGGTTACTTCCACCCC-3’ and *pvama1_R613* 5’-GCGTGGTGTGGGAGGCCC-3’, and nest 2 *pvama1_F29* 5’- GCAAACCAAATCGCTGCC-3’ and *pvama1_R598* 5’- GCCTCGGGGTCGAGCATCTCGTC-3’. The PCR for nest 1 was prepared as 1X of GoTaq Colorless Mastermix (Promega, Madison, USA), 25 mM of MgCl_2_, 10 µM of each primer and 2 µL of genomic DNA; for a final PCR volume of 25 µL. The PCR cycle was 94 ºC for 3 min, followed by 40 cycles of 94 ºC for 1 min, 46 ºC for 1 min and 72 ºC for 2.5 min, and a final extension at 72 ºC for 10 min [35].

The PCR for nest 2 of *pvama1* was prepared as 1X of GoTaq Colorless Mastermix (Promega, Madison, USA), 25 mM of MgCl_2_, 10 µM of each primer, and 3 µL of the previous PCR product; for a total PCR reaction volume of 37.5 µL. The PCR cycle was 94 ºC for 3 min, followed by 40 cycles of 94 ºC for 1 min, 57 ºC for 1 min and 72 ºC for 2.5 min, and a final extension at 72 ºC for 10 min [35].

The amplified products were purified using Illustra™ ExoProStar™ Enzymatic PCR and Sequencing Clean-up kit (GE Healthcare Life Sciences, Buckinghamshire, UK). A solution was prepared with of 2.5 µL of exonuclease, 25 µL of alkaline phosphatase and 472.5 µL of H_2_O, for a final volume of 500 µL. Afterwards, 5 µL of this solution was poured in each sample. Then, they were incubated at 37 ºC for 25 min and, subsequently, at 80 ºC for 20 min.

The purified products of *pvmsp1-19*, *pvdbpII* and *pvrbp1a* were sequenced using their own forward and reverse primers. For *pvama1* the following primers:

*pvama1_F142* 5’-AGAATTCCAGCTGGAAGATG-3’, *pvama1_F301* 5’-CGTAAAAATTTAGGAAACGCC-3’, *pvama1_F441* 5’-CCCCTGCAGCATATATAAAGAC-3’, *pvama1_R138* 5’-TTCCACTTCTGCATCTTCCCC-3’, *pvama1_R293* 5’-ACATTTTTGCTCAAATACACC-3’ and *pvama1_R442* 5’-GGGAAATCCCGGTCTACTTC-3’ were used to encompass the whole sequence [35]. The sequencing was performed in a 4-capillary electrophoresis genetic analyzer (Applied Biosystems model 3130xl) at Macrogen (Seoul, KOR).

The quality of pherograms was verified manually and incomplete and low-quality sequences were not further analysed. Geneious v10.1.3 (Biomatters Limited, NZ) was used to assemble, align, and edit the raw sequences to obtain consensus sequences for each gene fragment.

### Data analysis

The consensus sequences were aligned to the corresponding genes of Sal1, as follows: for *pvmsp1-19*, PVX_099980 (PlasmoDB); for *pvdbpII*, PVX_110810 (PlasmoDB); for *pvrbp1a-2*, AY501884 (GenBank) and for *pvama1*, PVX_092275 (PlasmoDB). DNA polymorphisms were analysed for the four gene fragments. The number of segregating sites (S), singletons (Si), parsimony sites (Pa), the average number of nucleotide differences (k), number of haplotypes (H), haplotype diversity (Hd), nucleotide diversity (π), and their corresponding standard deviations (SD) were calculated with DnaSP v5.1 software [39].

The genetic relationship between haplotypes, of *pvdbpII*, *pvrbp1a-2* and *pvama1* and how they were distributed in the locations, were constructed using Median Joining Network algorithm in NETWORK v5.0.0.1 software.

Tajima's D and Fu & Lì’s D* and F* were calculated to test the neutral theory of evolution. Positive or negative significant values of both tests reject the neutral theory of evolution, suggesting the influence of natural selection [38]. The McDonald-Kreitman test was used to analyse synonymous and nonsynonymous variation. Under neutrality, the ratio of replacement to synonymous fixed substitutions between groups should be the same as the ratio of replacement to synonymous polymorphisms within groups [39].

Wright’s fixation statistics (F_ST_) indexes were calculated to determine the degree of differentiation between the *P. vivax* from Ecuador and from other geographic origins, based on *pvdbpII*, *pvrbp1a-2* and *pvama1* invasion genes. F_ST_ values range from 0 to 1, high values indicate considerable degree of differentiation between parasite populations. The respective analyses were completed using the Arlequin v3.5.2.2 software.

A total of 638 sequences for *pvdbpII* from other geographic sites were obtained from the NCBI GenBank. For Brazil (BRZ), n = 122: EU812839-EU812960 [34]. For Colombia (COL), n = 17: U50575-U50591 [33]. For India (IND), n = 100: FJ491142-FJ49124 (Prajapati and Joshi, unpublished). For Iran (IRN), n = 19: EU860428-EU860438 [40], KF318358-KF318359 [41], and KF791921-KF791926 [42]. For South Korea (SK), n = 15: JN989472-JN989484 [43], and AF215737-AF215738 [44]. For Mexico (MEX), n = 35: KP759780-KP759814 [37]. For Papua Nueva Guinea (PNG), n = 200: AF289480-AF291096 [45], AY970837-AY970925 [46], and AF469515-AF469602 [47]. For Sri Lanka (SLK), n = 100: GU143914-GU144013 [48]. For Thailand (THL), n = 30: EF219451-EF379135 [49].

A total of 437 sequences were obtained for *pvama1*. For India (IND), n = 8: EF025187-EF025197 [50]. For Papua Nueva Guinea (PNG), n = 102: KC702402-KC702503 [51]. For Sri Lanka (SLK), n = 23: EF218679-EF218701 [52]. For Thailand (THL), n = 231: FJ784891-FJ785121 [53]. For Venezuela (VNZ), n = 73: EU346015-EU346087 [35].

Polymorphic residues were mapped on three-dimensional structural models for PvDBPII (4NUV) and PvAMA1 (1W81) using Discovery Studio Visualizer v16.1.0. B-cell epitope predictions for PvAMA1 were based on *P. vivax* Sal1 (PVX_092275) using BepiPred-2.0: Sequential B-Cell Epitope Predictor [54]. An epitope threshold of 0.5 with a length of at least 5 amino acids long was applied, as suggested in a previous study [55].

## Results

### **Genetic polymorphism of *P. vivax *merozoite invasion genes**

This study analysed *P. vivax* samples from two geographically and ecologically separate malaria endemic regions of Ecuador on both sides of the Andes Mountains. A *pvmsp1-19* gene fragment of 294 bp (codons 1646–1743) was analysed from a total of 72 samples. Only one nonsynonymous change was detected at codon 1660 Arg/Trp (TGG –AGG), belonging to sample Pv066 (Eastern Amazon) (Table 1). A gene fragment of 909 bp (codons 224–525) was analysed from a total of 50 samples for *pvdbpII*. Sequence analysis using the Sal1 strain as reference showed 44 polymorphisms, 9 were synonymous and 35 nonsynonymous. Ten of these 35 nonsynonymous mutations have been reported frequently worldwide (R308S, K371E, D384G, E385K, K386N, R390H, N417K, L424I, W437R and I503K) [56]. In addition, only one trimorphic site was detected (I516F/M), whereas the others were dimorphic. Interestingly, sample Pv009 exhibited an insertion (CTA) that coded for a Leu (L475) and was the only sequence with a change in residue F306L. Diversity analysis indicated a total number of 28 haplotypes (H) among the 50 samples analysed, which represented a nucleotide diversity (π) of 0.00736, and a haplotype diversity (Hd) of 0.940 (Table 1).

**Table 1:** Comparison of *P. vivax* merozoite gene diversity among Ecuadorian samples, 2012–2015

Gene	S ^a^	Si ^b^	Pa ^c^	K ^d^	H ^e^	Hd ^f^ (SD)	π ^g^ (SD)
*pvmsp1-19* (N = 72)	1	1	0	0.028	2	0.028 (0.027)	0.00009 (0.00009)
*pvdbpII* (N = 50)	45	26	19	6.667	28	0.940 (0.019)	0.00736 (0.00059)
*pvrbp1a-2* (N = 70)	53	32	21	6.363	31	0.924 (0.018)	0.00747 (0.00075)
*pvama1* (N = 73)	33	4	29	6.986	16	0.836 (0.031)	0.00439 (0.00033)

^a^S: Polymorphic sites

^b^Si: Singletons

^c^Pa: Parsimony sites

^d^K: The average of nucleotide differences

^e^H: Number of haplotypes

^f^Hd: Haplotype diversity

^g^π: Nucleotide diversity

A *pvrbp1a-2* gene fragment of 852 bp (codons 351–634) was analysed from a total of 70 samples. Fifty-six polymorphic sites were detected, 9 were synonymous and 47 nonsynonymous. Five trimorphic sites were observed (N352Y/H, E360K/G, D388G/E, G599L/E and Q625R/P), while the rest were dimorphic. The analysis showed 31 haplotypes (H), which represented, a nucleotide diversity (π) of 0.00747 and a haplotype diversity (Hd) of 0.924 (Table 1).

The entire CDS of the *pvama1* gene, with a total length of 1593 bp (codons 1-531), was analysed from 73 samples. Nucleotide sequence comparison with the Sal1 sequence revealed 33 SNPs, 6 of which were synonymous and 27 nonsynonymous. Furthermore, 18 of the 33 found in Ecuadorian isolates were frequent polymorphic residues found globally (G42V, R66K, D107A, R112T, K120R, N130K, N132D, L140I, A141E, E145A, E189K, K190Q, P210S, E277K, K352N, Q380K, L384P y E385D) [51]. Diversity indexes revealed a total number of 16 haplotypes (H), which represented a nucleotide diversity (π) of 0.00439, and a haplotype diversity (Hd) of 0.836 (Table 1).

### **Diversity and genetic differentiation of *P. vivax *invasion genes**

**Fig. 2:**
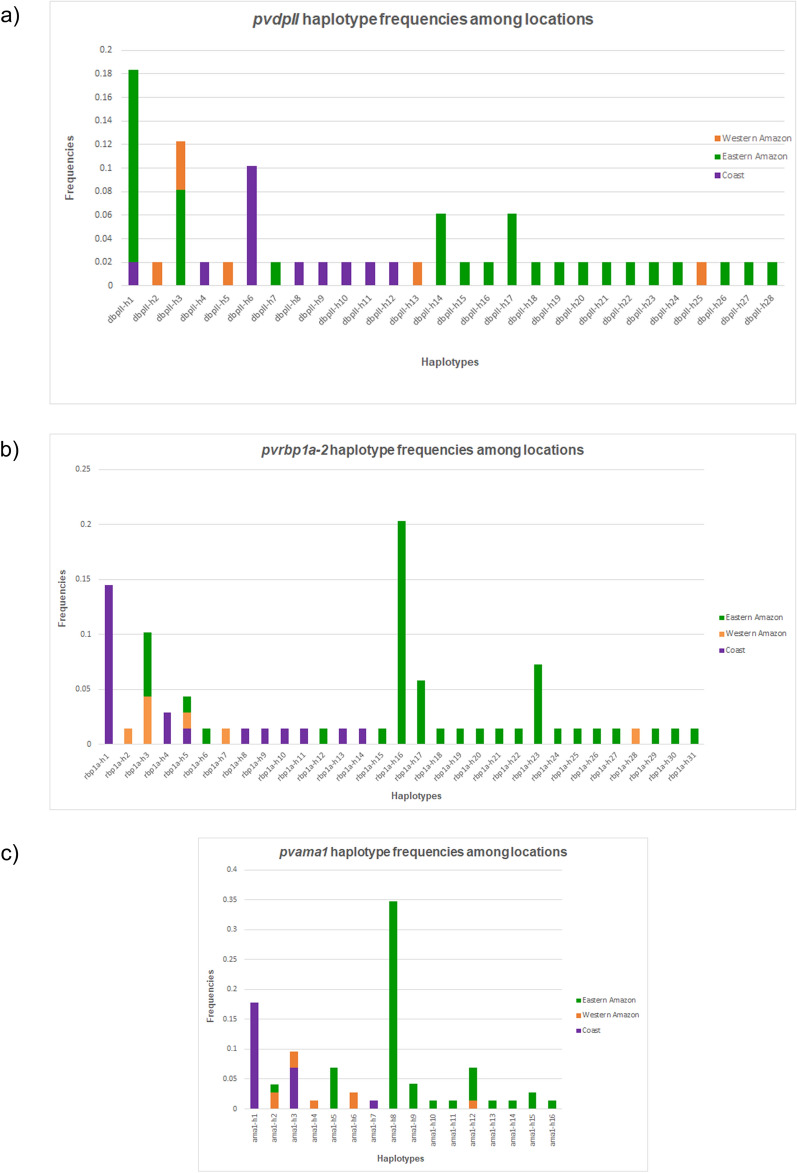
Haplotype frequencies of *Plasmodium vivax* genes encoding merozoite surface proteins in Ecuador, 2012–2015. **a** *pvdbpII* haplotypes frequencies in the study sites. **b** *pvrbp1a-2* haplotypes frequencies in the study sites. **c** *pvama1* haplotypes frequencies in the study sites

There were 28 different haplotypes of *pvdbpII* based on the nucleotide sequence analysis of CDS for this receptor binding domain. Eastern Amazon was the locality that had most of the haplotypes identified (n = 17), followed by Coast (n = 8) and Western Amazon (n = 5) (Fig. 2). Remarkably, *pvdbpII* haplotypes were generally distinct among these locations as only 2 variants (*dbpII-h1* and *dbpII-h3*) were shared. For *pvrbp1a-2*, 31 haplotypes were detected, most of them distributed in Eastern Amazon (n = 20), followed by Coast (n = 9) and Western Amazon (n = 5) (Fig. 2). Only one *pvrbp1a-2* haplotype was shared within the three locations (*rbpa-h5*) and the variant (*rbp1a-h3*) was reported in both eastern and Western Amazon. A total of 16 haplotypes were identified. For *pvama1*, 16 haplotypes were detected. Most of th*e pvama1* haplotypes were located in the Eastern Amazon (n = 11), followed by Western Amazon (n = 5) and Coast (n = 3). Two variants (*ama1-h2* and *ama1-h12*) were localized within Eastern and Western Amazon, while the haplotype *ama1-h3* was shared between Coast and Western Amazon (Fig. 2).

**Fig. 3:**
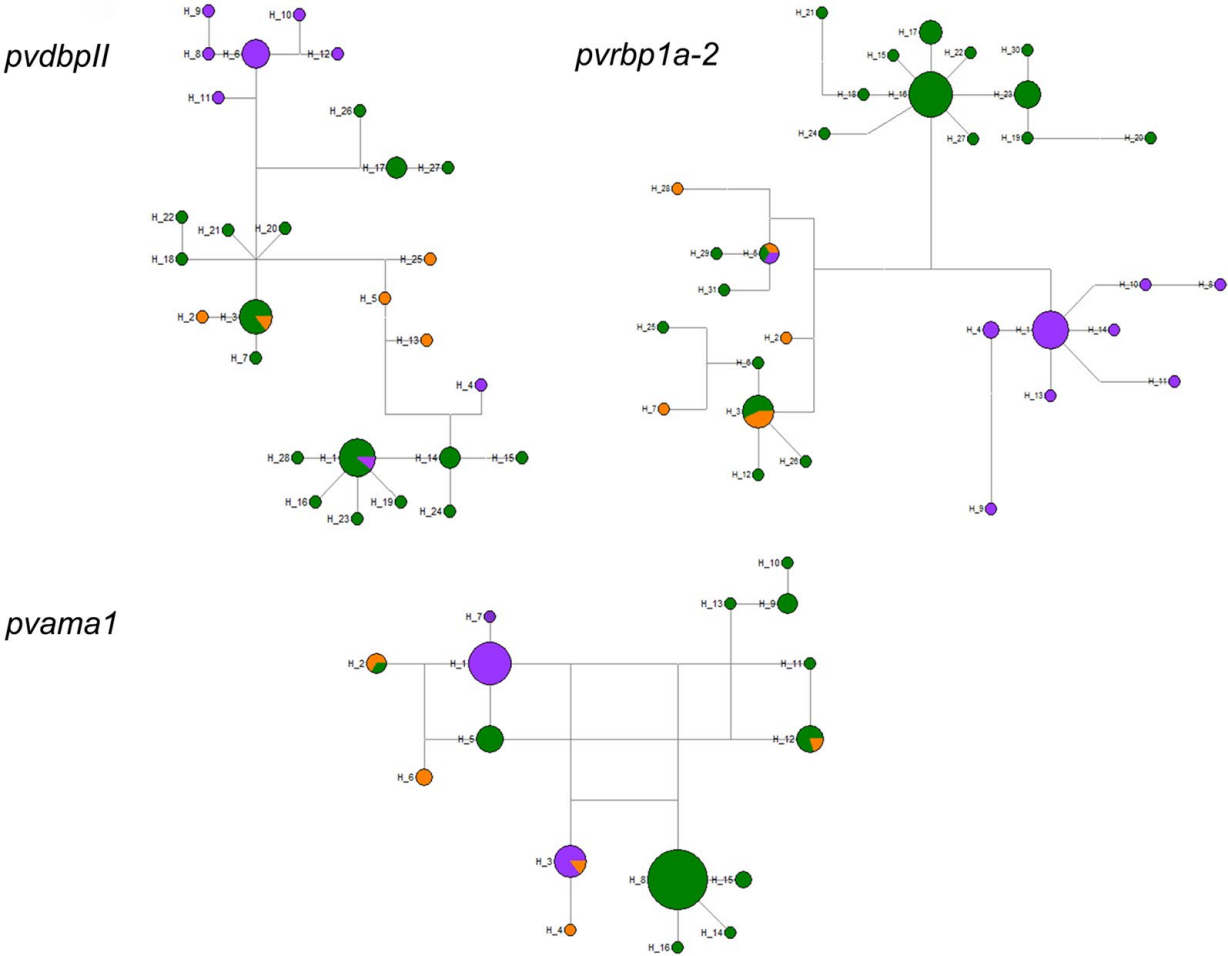
Haplotype networks of *Plasmodium vivax* genes encoding merozoite surface proteins in Ecuador, 2012–2015. The haplotype network for each marker is shown. Each circle represents one haplotype, and the circle size indicates frequency. Colours represent the geographical origin of the samples, Eastern Amazon (green), Western Amazon (orange) and Coast (purple)

Based on these data a haplotype network was constructed for *pvdbpII, pvrbp1a-2* and *pvama1*. In general, all haplotypes belonging to the Eastern Amazon were more related to those from Western Amazon. Not unexpectedly, haplotypes localized in Coast exhibited less relationship with the variants from the Amazon Region. While haplotypes are mixed in different locations, most haplotypes are restricted to a particular location and most locations have related haplotypes (Fig. 3).

In particular, analysis of the *pvdbpII* gene fragment showed the highest haplotype diversity in Western Amazon (0.933), while the highest value of nucleotide diversity was found in Eastern Amazon (0.00588). F_ST_ values showed moderate differentiation between parasites from the Eastern and Western Amazon (0.14257). In contrast, there was high differentiation between *P. vivax* from the Amazon Region and those from the Coast Region (Additional file 1: Table S1). In *pvrbp1a-2*, the highest haplotype diversity was found in Eastern Amazon (0.875), whereas nucleotide diversity value was higher in Western Amazon (0.00626). Moreover, F_ST_ values considered a great differentiation of the populations among three locations (F_ST_>0.25). However, the F_ST_ value was lower (0.30473) when comparing the populations of the Eastern and Western Amazon (Additional file 2: Table S2).

In the case of *pvama1*, the Western Amazon locality showed the highest values of haplotype and nucleotide diversity, 0.893 and 0.0693 respectively. Similar to *pvdbpII* and *pvrbp1a-2*, F_ST_ values demonstrated that parasites from the Amazon regions had lower genetic differentiation among themselves (0.27113). Likewise, genetic differentiation was more elevated among populations from the Coast and Western Amazon (0.35536), and between Coast and Eastern Amazon (0.39884) (Additional file 3: Table S3).

### **Genetic comparison of *P. vivax *merozoites genes from Ecuador with geographic sites around the world**

Six hundred and eighty eight sequences from different parts of the world were compiled for the comparative analysis of the ligand domain DBP region II (*pvdbpII*). The analysis of this region revealed that every country, except Mexico, had high levels of haplotype diversity (Hd), and ranged from 0.919 to 0.993. The major nucleotide diversity (π) was seen in Thailand with 0.01093 (Table 2). The largest haplotypic distance of Ecuadorian parasites measured by Fst values was with Iran (0.00936), India (0.05616) and Brazil (0.05981). In contrast, the closest distance was with samples from Colombia (Table 2).

**Table 2:** Diversity and genetic differentiation among diverse geographic populations of *P. vivax* using available sequences of the *pvdbpII* gene

Population (N)	S ^a^	π ^b^ (SD)	H ^c^	Hd ^d^ (SD)	F_ST_									
					ECU	BRA	COL	IND	IRN	SK	MEX	PNG	SLK	THL
ECU (50)	31	0.00903 (0.00067)	25	0.924 (0.022)	–									
BRA (122)	20	0.00817 (0.00033)	34	0.934 (0.012)	0.05981*	–								
COL (17)	14	0.00893 (0.00071)	16	0.993 (0.023)	0.19427*	0.15969*	–							
IND (100)	36	0.00878 (0.00050)	35	0.921 (0.017)	0.05616*	0.01283*	0.17868*	-						
IRN (19)	22	0.01004 (0.00113)	17	0.988 (0.021)	0.00936	0.00801	0.15142*	0.01529	–					
SK (15)	16	0.00553 (0.00063)	13	0.971 (0.039)	0.11660*	0.10658*	0.30625*	0.09359*	0.10616*	–				
MEX (35)	10	0.00400 (0.00077)	7	0.553 (0.092)	0.09361*	0.19368*	0.35432*	0.21595*	0.13550*	0.33474*	–			
PNG (200)	73	0.00971 (0.00033)	72	0.919 (0.012)	0.13954*	0.12336*	0.21038*	0.12734*	0.09830*	0.17530*	0.22687*	–		
SLK (100)	27	0.00973 (0.00053)	39	0.922 (0.014)	0.06443*	0.03066*	0.18452*	0.01917*	0.03126	0.08538*	0.22646*	0.13703*	–	
THL (30)	29	0.01093 (0.00054)	24	0.982 (0.014)	0.09646*	0.08425*	0.21381*	0.07510*	0.02140	0.13746*	0.20712*	0.13746*	0.10006*	–

^a^S: Polymorphic sites. ^b^π: Nucleotide diversity. ^c^H: Haplotypes. ^d^Hd: Haplotype diversity. *Statistically significant

A total of 510 sequences for the *pvama1* gene from six different countries were included in the comparative analysis. The haplotype diversity (Hd) observed in this gene ranged from 0.909 to 0.996. Ecuador was the country with the lowest haplotype diversity (0.836) (Table 3). The lowest haplotype distance of Ecuadorian *P. vivax* was found with Venezuela (Fst = 0.12935), indicating moderate differentiation between both nations. In contrast, the highest Fst value was observed when compared with Sri Lanka (0.48394) (Table 3).

**Table 3:** Estimation of the diversity and genetic differentiation among diverse geographic populations of *P. vivax* using available sequences of the *pvama1* gene

Population (N)	S ^a^	π ^b^ (SD)	H ^c^	Hd ^d^ (SD)	F_ST_					
					ECU	IND	PNG	SLK	THL	VNZ
ECU (73)	33	0.00465 (0.00035)	16	0.836 (0.031)	–					
IND (8)	27	0.00710 (0.00098)	6	0.929 (0.084)	0.35220*	–				
PNG (102)	44	0.00804 (0.00021)	87	0.996 (0.002)	0.37766*	0.11229*	–			
SLK (23)	34	0.00674 (0.00066)	15	0.949 (0.028)	0.48394*	0.24452*	0.25450*	–		
THL (231)	57	0.00834 (0.00018)	96	0.934 (0.012)	0.29150*	0.10716*	0.19695*	0.19117*	–	
VNZ (73)	29	0.00664 (0.00016)	18	0.909 (0.016)	0.12935*	0.20635*	0.25816*	0.25052*	0.15506*	–

^a^S: Polymorphic sites

^b^π: Nucleotide diversity

^c^H: Haplotypes

^d^Hd: Haplotype diversity

*Statistically significant

### **Natural selection in *pvdbpII *and *pvama1***

Tests for neutrality were applied to determine if the allelic variants from Ecuadorian endemic locations may have resulted from potential selective forces. Fu & Li’s D* and F* neutrality tests, for the *pvdbpII* region revealed significant values (p < 0.05) of − 2.99684 and − 2.77579, respectively. Tajima´s D and McDonald-Kreitman test were not statistically significant, which indicated no departure from neutrality for the pvdpbII region (Table 4). On the other hand, *v*alues of Fu & Li’s D* (− 3.48451) and F* (− 1.50687) were highly significant for the *pvrbp1a-2* gene fragment (p < 0.02), while Tajima´s D value was not significant (Table 4).

**Table 4:** Natural selection of *P. vivax* genes encoding merozoite proteins

Gene	Fu & Li’s test		Tajima’s D test	McDonald-Kreitman test		
	D* (P value)	F* (P value)	Tajima’s D (P value)	Within spp. (S ^a^/NS ^b^)	Between spp. (S/NS)	P value
*pvdbpII* (N = 50)	− 2.99684 (P < 0.05) *	− 2.77579 (P < 0.05) *	− 1.15380 (P > 0.10)	9/34	56/105	0.098432
*pvrbp1a-2* (N = 70)	− 3.80015 (P < 0.02) *	− 3.48451 (P < 0.02) *	− 1.50687 (P > 0.10)	–	–	–
*pvama1* (N = 73)	0.81125 (P > 0.10)	0.64509 (P > 0.10)	0.09268 (P > 0.10)	6/27	130/85	0.000007*

^a^Synonymous substitution

^b^Nonsynonymous substitution

*Statistically significant

In the *pvama1* CDS, results for the Fu & Li’s D*, F*, and Tajima´s D were not significant. However, the result of the McDonald-Kreitman test revealed a highly significant value (p = 0.000007), which demonstrated a departure from neutrality in *pvama1* gene of Ecuadorian *P. vivax* (Table 4).

To determine the most variable region of *pvama1*, genetic diversity was evaluated independently in the three domains. The analysis revealed that domain I (DI) was the most diverse in the whole CDS and domain III (D3) showed the lowest values (Additional file 4: Table S4). The values of Fu & Li D*, and F* neutrality tests and Tajima’s D in DI were nor significant. On the other hand, McDonald-Kreitman test resulted in a highly significant value (p = 0.009933) (Table 5). In D II, the values of Fu & Li D*, F* and Tajima’s were not significant, but they were for the McDonald-Kreitman test (p = 0.000707). The results of the McDonald-Kreitman test suggested departure from neutrality in both domains (Table 5).

**Table 5:** Natural selection on *pvama1* from Ecuadorian samples, 2012–2015

Domains	Fu & Li’s test		Tajima’s D test	McDonald-Kreitman test		
	D* (P value)	F* (P value)	Tajima’s D (P value)	Within spp. (S^a^/NS^b^)	Between spp. (S/NS)	P value
Whole gene (N = 73)	0.81125 (P > 0.10)	0.64509 (P > 0.10)	0.09268 (P > 0.10)	6/27	130/85	0.000007*
DI	1.26773 (P > 0.10)	1.20698 (P > 0.10)	0.56456 (P > 0.10)	5/14	46/30	0.009933*
DII	0.51261 (P > 0.10)	0.47490 (P > 0.10)	0.18210 (P > 0.10)	0/8	30/16	0.000707*
DIII	− 1.35328 (P > 0.10)	− 1.51472 (P > 0.10)	− 1.15994 (P > 0.10)	1/3	20/9	0.09198

^a^Synonymous substitution

^b^Nonsynonymous substitution

*Statistically significant

### Analysis of polymorphisms associated with neutralizing antibody epitopes in PvDBPII and PvAMA1

**Fig. 4:**
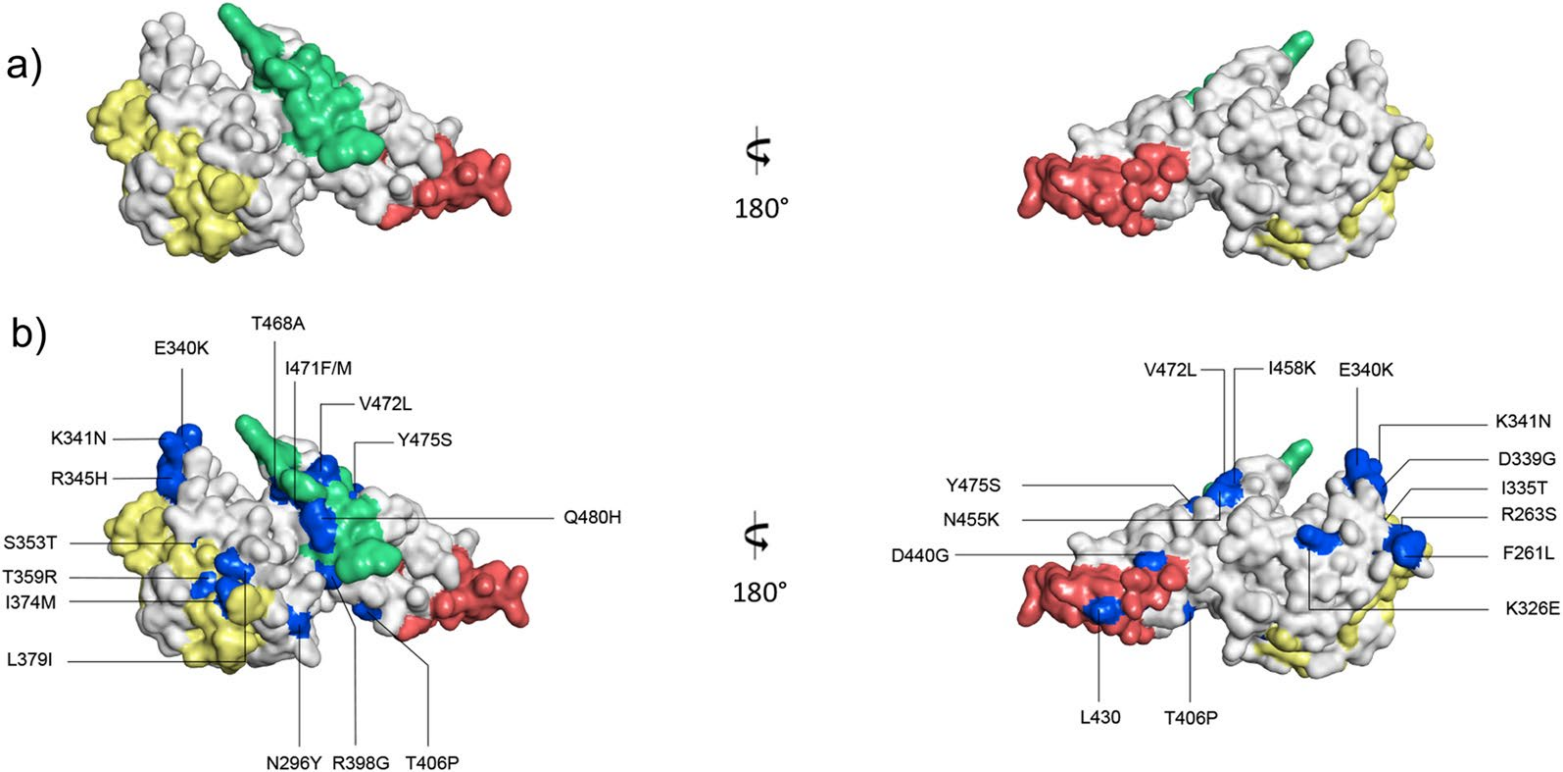
Three-dimensional structure of *Plasmodium vivax* DBPII showing the polymorphisms and the neutralizing mAb epitopes. **a** Solvent-accessible surface representation of two opposite sides of the PvDBPII three-dimensional structure. Broadly neutralizing epitopes of murine monoclonal antibodies 2D10 and 2H2 are shown in light red whereas the 2C6 epitope in light green. Epitopes of human neutralizing monoclonal antibodies (053054 and 092096) are shown in yellow. **b** Solvent-accessible surface representation of two opposite sides of the PvDBPII three-dimensional structure. Polymorphic residues are shown in blue and labelled

Recent studies using inhibitory monoclonal antibodies (mAbs) have shown conserved epitopes that elicit a broadly neutralizing response and may confer protective immunity against different strains of *P. vivax* [57, 58]. Therefore, the presence of polymorphic residues in epitopes of three broadly neutralizing murine antibodies (bNAbs) 2D10, 2H2 y 2C6, and of two human mAbs (053054 and 092096) in PvDBPII were evaluated. One insertion at position 430 (L430) and two amino acid changes (A435G and D440G) were observed within 2D10/2H2 epitopes (residues 413–441). Moreover, five substitutions (T468A, I471F/M, V472L, Y475S and Q480H) were present in 2C6 epitope (residues 465–485) (Fig. 4).

The discontinuous conformational epitope for mAb 053054 is composed of amino acids 264–281, 356–372 and 249, whereas 092096 binds to the epitope comprised of residues 270–289, 355–375, 249 and 219 [59]. According to this, four polymorphic residues (T359R, M361L and N372K and I374M) were found for both mAbs epitopes (Fig. 4).

**Fig. 5:**
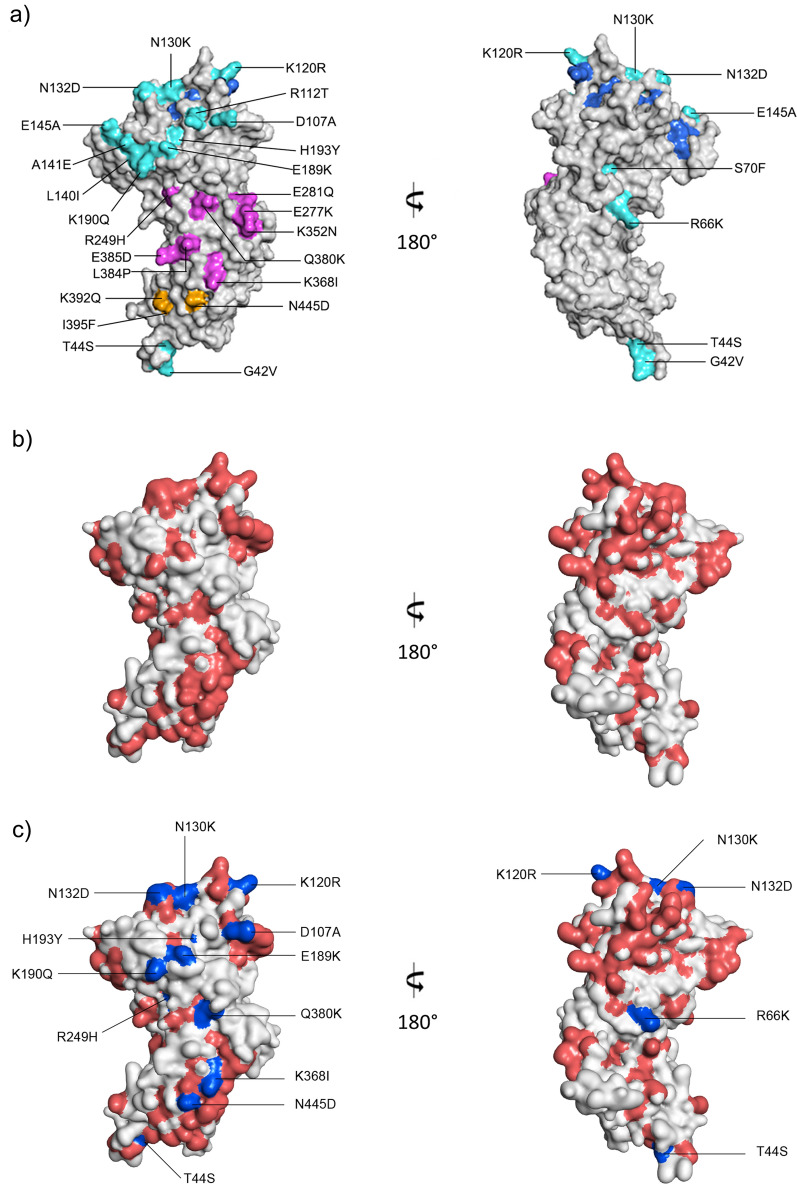
Three-dimensional structure of *Plasmodium vivax* AMA1 with the polymorphisms and the predicted B-cell epitopes mapped **a** Solvent-accessible surface representation of the active (left) and silent face (right) of the PvAMA1 three-dimensional structure. Polymorphic residues are coloured according to location: DI (cyan), DII (magenta) and DIII (orange). Hydrophobic ligand binding cleft residues are shown in dark blue. **b** Solvent-accessible surface representation of two opposite sides of the PvAMA1 three-dimensional structure. Residues of the linear B-cell epitopes are shown in light red. **c** Solvent-accessible surface representation of two opposite sides of the PvAMA1 three-dimensional structure. Polymorphic residues are shown in light blue

Twenty-two predicted linear B-cell epitopes were found across PvAMA1, with 9 containing at least one polymorphic site. Among the 27 nonsynonymous substitutions found at PvAMA1, 15 sites are located within B-cell epitopes (Additional file 5: Table S5). Mutation sites inside predicted B cell epitopes were mapped on a three-dimensional structure of PvAMA1 (Fig. 5).

## Discussion

In Ecuador, malaria has been a major public health problem over the last decades. Between 2001 and 2015 documented clinical cases decreased by more than 99%, leading the country towards the elimination phase. Nevertheless, several periodic outbreaks of malaria have occurred in the endemic regions of the country, and from 2015 the number of cases has increased, especially affecting some of the poorest inhabitants in Ecuador’s endemic areas. It is not yet certain whether the increasing incidence of vivax malaria is due to destabilization of local transmission patterns or new immunologically distinct parasite strains from different endemic regions.

Different biological and socioeconomic factors contribute, in several ways, to the maintenance of *P. vivax* diversity, including relapses (caused by hypnozoites), early gametocyte development, asymptomatic infections and the late diagnosis and treatment [53, 56]. However, the main mechanisms to generate and maintain diversity in the merozoite invasion genes occur from natural selection caused by the host immune responses and the diversity created by natural meiotic recombination among different variants of parasites inside the mosquito’s midgut [34, 60, 61].

In this study, diversity of invasion ligands was analyzed in Ecuadorian *P. vivax*. A novel substitution was found in *pvmsp1-19*. There were previously only three reports of mutations in this region: D1706E [62], K1709E [63] and N1692K [64]. Overall, *pvmsp1-19* was highly conserved in Ecuadorian *P. vivax* parasites, which is consistent with previous studies reported worldwide [37, 63–66]. MSP1_19_ is highly preserved [37, 64, 67]. It is presumed the highly conserved nature of MSP1_19_ is due to an important functional role, especially in the food vacuole formation [68].

The analysis of *pvdbpII* revealed 35 nonsynonymous substitutions, of which 10 (R308S, K371E, D384G, E385K, K386N, R390H, N417K, L424I, W437R y I503K) have been widely reported in different endemic regions [56]. In addition, polymorphic residues in the positions N417K, W437R and I503K, found in most of the Ecuadorian samples, have been considered as targets of binding inhibitory antibodies (Fig. 4). This suggests that these mutations, alone or in combination, could affect the binding efficiency of the antibodies [69, 70]. Interestingly, residue F306L, which was found in sample Pv009 (Coast), had only been reported previously in isolates from Asia [40, 49, 71]. The same sample had a CTA codon insertion, which coded for a leucine between amino acid 474 and 475. This insertion has been found in samples from India, Iran, Indonesia, Thailand, and Brazil [42].

A surprisingly high level of genetic diversity was discovered in Ecuadorian parasites, and *pvdbpII* was the highest in terms of haplotype diversity (0.940). This finding is consistent with several studies in different endemic countries in which similar levels of diversity have been found [33, 34, 48, 71, 72]. The results indicate that *pvdbpII* is consistently polymorphic at a national and worldwide scale, although there was no evidence of departure from neutrality in this region. However, significant values in Fu & Li’s D* and F* could be suggesting selective pressure. This would imply that a mutation involved in a more efficient way to evade the host immune response occurred within this region, thereby increasing parasite fitness. Such mutations provide an adaptative advantage in individuals to increase frequency in the population. These results are similar to those obtained in Mexico by González-Cerón and collaborators in 2015, although studies in other endemic regions revealed evidence of balancing selection in *pvdbpII* ligand domain that is considered to be the target of anti-DBP protective immunity [33, 48, 49, 71–73].

PvDBPII inhibitory mAbs epitopes in Ecuadorian parasites revealed a lack of high-frequency polymorphisms. This is consistent with a previous study, in which DBPII sequences that represent global diversity were analyzed for retention of the epitopes for 2D10, 2H2, and 2C6 antibodies [57, 59]. Together, 2D10, 2H2, and 2C6 recognize broadly conserved epitopes and play a role as bNAbs that will be effective against different *P. vivax* strains [57]. Only four amino acid changes (T359R, M361L and N372K and I374M) were identified in human mAbs epitopes (053054 and 092096) (Fig. 4). A comparative analysis demonstrates that mutations T359R, N372K and I374M individually and in combination do not affect antibody binding to DBPII.

Some epitopes accumulate a greater number of polymorphisms in order to create immunologically-distinct B-cell epitopes, allowing the parasite to evade the acquired immune responses and lead to successful invasion of the reticulocytes. On the other hand, there are regions evolving neutrally. The contrast of selective pressures in *pvdbpII* has been previously reported for region IV [34, 74]. This may imply a balance between the high functional restriction, maintaining invariable structures due to the important role that DBP protein plays in reticulocyte binding and the search for diversity as a response against the pressure of the host’s immune system [74].

The analysis of *pvrbp1a-2* gene fragment revealed 9 synonymous substitutions and 47 nonsynonymous. Interestingly, sequences from four isolates (Pv009, Pv034, Pv057 and Pv066) were identical to the reference parasite Sal 1. *pvrbp1a-2* had the highest nucleotide diversity (0.00747) (Table 1). Previous studies in *pvrbp1a* have shown that the region between amino acids 435 and 777 is the most polymorphic of the whole protein, suggesting it as a target of antibody response [75, 76].

Neutrality tests carried out in this gene fragment showed highly significant values for Fu & Li’s D* and F*, but not significant for Tajima’s D value (Table 4). A mutation could have increased rapidly its frequency within the population and led to a decrease in variability in this fragment. In addition, McDonald-Kreitman test could not be performed to corroborate this result due to *P. knowlesi pvrbp1a* orthologous gene corresponded to a pseudogene highly degenerated (*ψpknbp1*) possibly due to an ancient recombination event [77]. The results in this study suggest that *pvdbpII*, *pvrbp1a-2* went through a selective sweep process as an adaptative advantage.

The analysis of the whole *pvama1* gene showed a total of 33 polymorphic sites, 6 were synonymous and 27 nonsynonymous (Fig. 5). Of these, 18 have been widely reported in many endemic countries [51]. Besides, 15 polymorphisms have been identified in predicted linear B-cell epitopes and might play an important role in immune evasion process (Additional file 5: Table S5). This is consistent with a previous study in which residues encountered at positions E145A, P210S, R249H, K352N and N445D have been overlapping B-cell epitopes [78]. These findings suggested that variations in amino acids could affect the protein structure, causing changes in charge and polarity, and helping the parasite escape from the host immune response [79]. In the same way, mutations Q380K and L384P, found in this study, have been previously suggested to be implicated in the PvAMA1 protein structure modification [61, 78].

Furthermore, the major number of polymorphic residues were on the active face of the PvAMA1 ectodomain, principally within domain I and II (Fig. 5). This suggests that this side of the protein is more exposed to the immune response [51]. In contrast, only four polymorphic residues (G42V, T44S, R66K and S70F) were located on the other side of this protein (silent face), which seems to have no relevance in the invasion process (Fig. 5) [79]. In addition, several polymorphic residues were grouped forming shield-type structure, which maintain preserved the RON2 gap union site. This could be a parasite’s mechanism to divert the immune response, accumulating mutations in non-relevant regions and thus keep highly conserved relevant structures for the invasive process [79] (Fig. 5).

Although values of Fu & Li’s D* and F* and Tajima’s D were non-significant, a slightly departure from neutrality was shown. Nevertheless, McDonald-Kreitman test determined a highly significant value (p = 0.000007), which evidenced the presence of balancing selection in this gene (Tables 4 and 5). These results were consistent with several investigations that found evidence of balancing selection, principally, in domains I [35, 51, 80, 81] and II [31, 61, 82]. Thus, the selective pressure by the human immune system causes that parasites keep mutations and maintain diversity in both domains to evade the immune responses.

The haplotype arrangement of *pvdbpII*, *pvrbp1a-2* and *pvama1* revealed a major number of variants in Eastern Amazon (Fig. 2). Despite the low and unstable transmission in endemic areas of Ecuador, several periodic outbreaks have been recently reported in the east of the country. This event could lead to an increase in parasite circulation and, therefore a high level of recombination. Most samples come from a border area where there is common cross border movement, explaining a higher diversity of *P. vivax* in this locality. It has been shown that genetic diversity, particularly in *pvdbpII*, is not related to malaria endemic levels, since similar patterns have been observed in areas with low and unstable transmission, such as parts of the Brazilian Amazon and Papua New Guinea [56].

Only a few haplotypes were shared among localities and most of them showed a greater genetic relationship according to the locality where they belong. Moreover, the variants found in the Western Amazon were more related to those from the Eastern Amazon, and both had less genetic similarity with the haplotypes from the Coast (Fig. 3). Similarly, F_ST_ analyses exhibited a high difference between parasites from the Coast Region and those from the Amazon Region (0.355–0.556). (Additional file 1: Table S1, Additional file 2: Table S2, Additional file 3: Table S3). These results are in accordance with previous reports using microsatellites in the same samples [83] that revealed important difference among Amazonian and Coastal parasites due to the geographical distance and the presence of geological barriers such as the Andes Mountain range. Although the diversity found in invasion ligand genes (*pvdbpII* and *pvama1*) in Ecuador was high, when comparing it to other parts of the world, the country had one of the lowest values (Tables 2 and 3).

Analysis of global *pvdbpII* sequences revealed low differentiation of parasite populations among countries, finding the same haplotypes in different countries, which is consistent with a previous study by De Sousa et al. [56], in which a global analysis of *pvdbpII* showed low differentiation of parasite populations among countries. Moreover, haplotypes found were all present in the eight different countries.

These findings confirm that merozoite antigenic diversity, rather than being influenced by geographic barriers, is more likely dependent on intrinsic characteristics of human populations, principally immune response [84]. Moreover, due to the differences in the endemicity spectrum, both recombination and natural selection (product of host immune system) seem to be differentially influenced between distinct geographical areas [56], which suggests that both mechanisms play a determinant role in the haplotype structure.

## Conclusions

The differences in diversity in the main *P. vivax* genes involved in reticulocyte invasion depend on the role that each play in this process. Possibly, the prolonged exposure of PvDBPII and PvRBP1a-2 to the host immune system contributed to maintain high diversity. PvAMA1, which seemed to be less exposed, has a moderate diversity. On the contrary, *PvMSP1-19* despite of being present from the initial attachment, is not fully exposed and, therefore, is a conserved region. *pvdbpII* and *pvama1* genetic diversity found in Ecuadorian *P. vivax* confirmed that diversity in these genes is not related to levels of endemicity. The major diversity of *P. vivax* in Ecuador was identified within the Amazonian localities possibly because of parasite migration from Peru in addition to locally circulating parasites and is in concordance with the increase in the number of infections in this area of the country. Natural selection behaves differently in each gene. Even more, acts differentially through them. *pvdbpII* and *pvrbp1a-2* were possibly affected by a selective sweeping process, whereas in *pvama1* the influence of balancing selection was evidenced, mainly in domains I and II .

### Supplementary information

**Additional file 1: Table S1.** Diversity and genetic differentiation in *pvdbpII* among Ecuadorian locations.

**Additional file 2: Table S2.** Diversity and genetic differentiation in *pvrbp1a-2* among Ecuadorian locations.

**Additional file 3: Table S3.** Diversity and genetic differentiation in *pvama1* among Ecuadorian locations.

**Additional file 4: Table S4.** Genetic diversity among *pvama1* domains in Ecuadorian samples.

**Additional file 5: Table S5.** Polymorphisms associated with predicted linear B-cell epitopes in PvAMA1 among Ecuadorian samples.

## Data Availability

The datasets generated and/or analysed during the current study are available from the corresponding author on reasonable request.

## Abbreviations

*P. vivax*: *Plasmodium vivax*
*pvmsp1-19*: Plasmodium vivax merozoite surface protein 1 carboxyl fragment of 19 kD
*pvdbpII*: Plasmodium vivax duffy binding protein region II
*pvrbp1a-2*: Plasmodium vivax reticulocyte binding protein 1a region 2
*pvama1*: Plasmodium vivax apical membrane antigen 1
DARC: Duffy antigen chemokine receptor
RON2: Rhoptry neck protein 2
RBC: Red blood cell
BRZ: Brazil
COL: Colombia
IND: India
IRN: Iran
SK: South Korea
MEX: Mexico
PNG: Papua New Guinea
SLK: Sri Lanka
THL: Thailand
VNZ: Venezuela
